# Extracting Association Patterns in Network Communications

**DOI:** 10.3390/s150204052

**Published:** 2015-02-11

**Authors:** Javier Portela, Luis Javier García Villalba, Alejandra Guadalupe Silva Trujillo, Ana Lucila Sandoval Orozco, Tai-hoon Kim

**Affiliations:** 1 Group of Analysis, Security and Systems (GASS), Department of Software Engineering and Artificial Intelligence (DISIA), Faculty of Information Technology and Computer Science, Office 431, Universidad Complutense de Madrid (UCM), Calle Profesor José García Santesmases, 9, Ciudad Universitaria, Madrid 28040, Spain; E-Mails: jportela@estad.ucm.es (J.P.); javiergv@fdi.ucm.es (L.J.G.V.); asilva@fdi.ucm.es (A.G.S.T); asandoval@fdi.ucm.es (A.L.S.O.); 2 Facultad de Ingeniería, Universidad Autónoma de San Luis Potosí (UASLP), Zona Universitaria Poniente, San Luis Potosí 78290, Mexico; 3 Department of Convergence Security, Sungshin Women's University, 249-1 Dongseon-dong 3-ga, Seoul 136-742, Korea

**Keywords:** anonymity, mixes, network communications, statistical disclosure attack

## Abstract

In network communications, mixes provide protection against observers hiding the appearance of messages, patterns, length and links between senders and receivers. Statistical disclosure attacks aim to reveal the identity of senders and receivers in a communication network setting when it is protected by standard techniques based on mixes. This work aims to develop a global statistical disclosure attack to detect relationships between users. The only information used by the attacker is the number of messages sent and received by each user for each round, the batch of messages grouped by the anonymity system. A new modeling framework based on contingency tables is used. The assumptions are more flexible than those used in the literature, allowing to apply the method to multiple situations automatically, such as email data or social networks data. A classification scheme based on combinatoric solutions of the space of rounds retrieved is developed. Solutions about relationships between users are provided for all pairs of users simultaneously, since the dependence of the data retrieved needs to be addressed in a global sense.

## 1. Introduction

When information is transmitted through the Internet, it is typically encrypted in order to prevent others from being able to view it. The encryption can be successful, meaning that the keys cannot be easily guessed within a very long period of time. Even if the data themselves are hidden, other types of information may be vulnerable. In the e-mail framework, anonymity concerns the senders “identity, receivers” identity, the links between senders and receivers, the protocols used, the size of data sent, timings, *etc*. Since [1] presented the basic ideas of the anonymous communications systems, researchers have developed many mix-based and other anonymity systems for different applications, and attacks on these systems have also been developed. Our work aims to develop a global statistical attack to disclose relationships between users in a network based on a single mix anonymity system.

## 2. Introducing Anonymous Communications

The infrastructure of the Internet was initially planned and developed to be an anonymous channel, but nowadays, it is well known that anybody can spy on it with different non-robust tools, like, for example, using sniffers and spoofing techniques. Since the Internet's proliferation and the use of some services associated with it, such as web searchers, social networks, webmail and others, privacy has become a very important research area, not just for security IT experts or enterprises. Connectivity and the enormous flow of information available on the Internet are a very powerful tool to provide knowledge and to implement security measures to protect systems.

Anonymity is a legitimate means in many applications, such as web browsing, e-vote, e-bank, e-commerce and others. Popular anonymity systems are used by hundreds of thousands people, such as journalists, whistle blowers, dissidents and others. It is well known that encryption does not guarantee the anonymity required for all participants. Attackers can identify traffic patterns to deduce who, when and how often users are in communication. The communication layer is exposed to traffic analysis, so it is necessary to anonymize it, as well as the application layer that supports anonymous cash, anonymous credentials and elections.

Anonymity systems provide mechanisms to enhance user privacy and to protect computer systems. Research in this area focuses on developing, analyzing and executing anonymous communication networks attacks.

Two categories for anonymous communication systems are commented on below: high latency systems and low latency systems. Both systems are based on Chaum's proposal [1] that introduced the concept of mixing.

- High latency anonymity systems aim to provide a strong level of anonymity and are oriented to limited activity systems that do not demand quick responses, such as email systems. These systems are message-oriented systems.
- Low latency anonymity systems can be used for interactive traffic, for example web applications, instant messaging and others. These systems are connection-based systems and are used to defend from a partial attacker who can compromise or observe just a part of the system. According to its nature, these systems are more susceptible to timing attacks and traffic analysis attacks. The majority of these systems depend on onion routing [2] for anonymous communication.

In low latency communication systems, an attacker only needs to observe the flow of the data stream to link sender and receptor users. Traditionally, in order to prevent this attack, dummy packets are added and delays incorporated into stream data to make the traffic between users uniform. The previously mentioned scenario can be useful for passive attackers that do not insert timing partners into the traffic to compromise anonymity. An active attacker can control routers of the network. Timing attacks are one of the main challenges in low latency anonymous communication systems. These attacks are closely related to traffic analysis in mix networks.

Traffic analysis techniques belong to the family of methods to infer information from the patterns in a communication system. Even when communication content has been ciphered, information routing needs to be sent clearly for routers to know the next package's destination in the network. Every data packet traveling on the Internet contains the node addresses of sending and recipient nodes. Therefore, it is well understood that, actually, no packet can be anonymous at this level.

### 2.1. Mixes and the Mix Network Model

Mixes are considered the base for building high latency anonymous communication systems. In network communications, mixes provide protection against observers hiding the appearance of messages, patterns, length and links between senders and receivers. Chaum [1] introduced also the concept of anonymous email. Their model suggested hiding the correspondence between senders and receivers encrypting messages and reordering them through a path of mixes before relaying them to their destinations. The set of the most likely receivers is calculated for each message in the sequence, and intersection of sets will make it possible to know who the receiver of the stream is.

**Figure 1.**
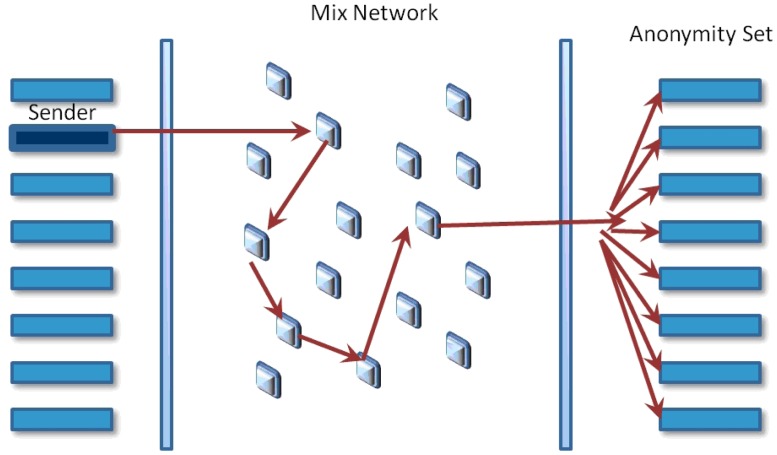
Mix network model.

A mix networks aims to hide the correspondences between the items in its input and those in its output, changing the incoming packets appearance through cryptographic operations (see Figure 1). The anonymity set is the set of all possible entities who might execute an action. The initial process in order for Alice send a message to Bob using a mix system is to prepare the message. The first phase is to choose the message transmission path; it has a specific order for iteratively sending messages before arriving at its final destination. It is recommended to use more than one mix in every path for improving system security. The next phase is to utilize the public keys of the chosen mixes for encrypting the message in the inverse order that they were chosen. Therefore, the public key of the last mix initially encrypts the message, then the next one before the last one, and finally, the public key of the first mix will be used. Every time a message is encrypted, a layer is built, and the next node address is included. This way, when the first mix gets a message prepared, this will be decrypted with its correspondent private key and will get the next node address.

An observer or an active attacker should not be able to find the link between the bit pattern of encoded messages arriving at the mix and decoded messages departing from it. Appending a block of random bits at the end of the message has the purpose of making messages uniform in size.

### 2.2. ISDN-Mixes

The first proposal for the practical application of mixes [3] showed the way a mix-net could be used with ISDN lines to anonymize a telephone user's real location. The origin of this method took into account the fact that mixes in their original form imply a significant data expansion and significant delays, and therefore, it was often considered infeasible to apply them to services with higher bandwidth and real-time requirements. The protocol tries to defeat these problems.

### 2.3. Remailers

The first Internet anonymous remailer was developed in Finland and was very simple to use. A user added an extra header to the e-mail pointing out its final destination: an email address or a Usenet newsgroup. A server receives messages with embedded instructions about where to send them next without revealing their origin. All standard-based email messages include the source and transmitting entities at the headers. The full headers are usually eliminated. The application replaces the original email's source address with the remailer's address.

Babel [4], Mixmaster [5] and Mixminion [6] are some others anonymous communication designs. The differences between systems will not be addressed in our work. We centered only on senders and receivers active in a period of time, and we do not take into account message reordering, because this does not affect our attack. Onion routing [2] is another design used to provide low latency connection for web browsing and other interactive services. It is important to specify that our method does not address this kind of design; they can be treated by short-term timing or packet counting attacks [7].

## 3. The Family of Mix Systems Attacks

The attacks against mix systems are intersection attacks and aim to reduce the anonymity by linking senders with the messages that they send, receivers with the messages that they receive or linking senders with receivers. Attackers can derive relations of frequency through observation of the network, compromising mixes or keys, delaying or altering messages. They can deduce the messages' most probable destinations through the use of false messages sent to the network and using this technique to isolate target messages and to derive their properties. Traffic analysis belongs to a family of techniques used to deduce pattern information in a communication system. It has been proven that cipher by itself does not guarantee anonymity. See [8] for a review of traffic analysis attacks.

### 3.1. The Disclosure Attack

In [9], Agrawal and Kesdogan presented the disclosure attack, an attack centered on a single batch mix, aiming to retrieve information from a particular sender, called Alice. The attack is global, in the sense that it retrieves information about the number of messages sent by Alice and received by other users, and passive, in the sense that attackers cannot alter the network, for example, by sending false messages or delaying existent messages.

It is assumed that Alice has exactly m recipients and that Alice sends messages with some probability distribution to each of her recipients; also that she sends exactly one message in each batch of b messages. The attack is modeled considering a bipartite graph G. Through numerical algorithms, disjoint sets of recipients would be identified to reach, through intersection, the identification of Alice recipients. The authors use several strategies in order to estimate the average number of observations for achieving the disclosure attack. The assumptions are: (i) Alice participates in all batches; and (ii) only one of Alice's peer partners is in the recipient set of all batches. This attack is computationally expensive, because it takes an exponential time analyzing the number of messages to identify a mutually disjoint set of recipients. The main bottleneck for the attacker derives from an NP-complete problem when it is applied to big networks. The authors claim the method performs well on very small networks.

### 3.2. Statistical Disclosure Attacks

In [10], Danezis presents the statistical disclosure attack, maintaining some of the assumptions made in [9]. In the statistical disclosure attack, recipients are ordered in terms of probability. Alice must demonstrate consistent behavior patterns in the long term to obtain good results. The Statistical Disclosure Attack (SDA) requires less computational effort by the attacker and gets the same results. The method tries to reveal the most likely set of Alice's friends using statistical operations and approximations.

Statistical disclosure attacks when threshold mixing or pool mixing are used are treated also in [11], maintaining the assumptions of precedent articles, that is, focusing on one user, Alice, and supposing that the number of recipients of Alice is known. Besides, the threshold parameter B is also supposed to be known. One of the main characteristics of intersection attacks counts on a fairly consistent sending pattern or a specific behavior of anonymous network users.

Mathewson and Dingledine in [12] make an extension of the original SDA. One of the more significant differences is that they regard real social networks to have scale-free network behavior and also consider that such behavior changes slowly over time. The results show that increasing message variability makes the attack slow by increasing the number of output messages; assuming all senders choose with the same probability all mixes as entry and exit points and the attacker is a partial observer of the mixes.

Two-sided statistical disclosure attacks [13] use the possibilities of replies between users to make the attack stronger. This attack assumes a more realistic scenario, taking into account the user behavior on an email system. Its aim is to estimate the distribution of contacts of Alice and to deduce the receivers of all of the messages sent by her. The model considers N as the number of users in the system that send and receive messages. Each user n has a probability distribution Dn of sending a message to other users. At first, the target, Alice, is the only user that will be modeled as replying to messages with a probability r. An inconvenient detail for applications on real data is the assumption that all users have the same number of friends and send messages with uniform probability.

Perfect matching disclosure attacks [14] try to use simultaneous information about all users to obtain better results related to the disclosing of the Alice set of recipients. This attack is based on graph theory, and it does not consider the possibility that users send messages with different frequencies. An extension proposal considers a normalized SDA.

Danezis and Troncoso [15] present a new modeling approach, called Vida, for anonymous communication systems. These are modeled probabilistically, and Bayesian inference is applied to extract patterns of communications and user profiles. The authors developed a model to represent long-term attacks against anonymity systems. Assume each user has a sending profile, sampled when a message is to be sent to determine the most likely receiver. Their proposal includes: (1) the Vida black-box model representing long-term attacks against any anonymity systems. Bayesian techniques are used to select the candidate sender of each message: the sender with the highest *a posteriori* probability is chosen as the best candidate. The evaluation includes a very specific scenario considering the same number of senders and receivers. Each sender is assigned to five contacts randomly, and everyone sends messages with the same probability.

In [16], a new method to improve the statistical disclosure attack, called the hitting set attack, is introduced. Frequency analysis is used to enhance the applicability of the attack, and duality checking algorithms are also used to resolve the problem of improving the space of solutions. Mallesh and Wright [17] introduces the reverse statistical disclosure attack. This attack uses observations of all users sending patterns to estimate both the targeted user's sending pattern and her receiving pattern. The estimated patterns are combined to find a set of the targeted user's most likely contacts.

In [18], an extension to the statistical disclosure attack, called SDA-2H, is presented, considering the situation where cover traffic, in the form of fake or dummy messages, is employed as a defense.

Perez-Gonzalez *et al*. [19] presents a least squares approximation to the SDA, to recover users' profiles in the context of pool mixes. The attack estimates the communication user partners in a mix network. The aim is to estimate the probability of Alice sending a message to Bob; this will derive sender and receiver profiles applicable for all users. The assumptions are: the probability of sending a message from a user to a specific receiver is independent of previous messages; the behavior of all users are independent from one other; any incoming message in the mix is considered *a priori* sent by any user with a uniform probability; and the parameters used to model the statistical behavior do not change over time.

In [20], a timed binomial pool mix is used, and two privacy criteria to develop dummy traffic strategies are taken into account: (i) increasing the estimation error for all relationships by a constant factor; and (ii) guaranteeing a minimum estimation error for any relationship. The model consists of a set of N senders exchanging messages with a set of M receivers. To simulate the system, consider the same number of senders and receivers and assume users send messages with the same probability. Other work also based on dummy or cover traffic is presented in [21]. This assumes users are not permanently online so, so they cannot send cover traffic uniformly. They introduce a method to reveal Alice's contacts with high probability, addressing two techniques: sending dummy traffic and increasing random delays for messages in the system.

Each one of the previous works has assumed very specific scenarios, but none of them solves the problems that are presented by real-world data. In order to develop an effective attack, the special properties of network human communications must be taken into account. Researchers have hypothesized that some of these attacks can be extremely effective in many real-world contexts. Nevertheless, it is still an open problem under which circumstances and for how long of an observation these attacks would be successful.

## 4. Framework and Assumptions

This work addresses the problem of retrieving information about relationships or communications between users in a network system, where partial information is obtained. The information used is the number of messages sent and received by each user. This information is obtained in rounds that can be determined by equally-sized batches of messages, in the context of a threshold mix, or alternatively by equal length intervals of time, in the case that the mix method consists of keeping all of the messages retrieved at each time interval and then relaying them to their receivers, randomly reordered.

The basic framework and assumptions needed to develop our method are the following:

- The attacker knows the number of messages sent and received by each user in each round.
- The round can be determined by the system (batches) in a threshold mix context or can be based on regular intervals of time, where the attacker gets the aggregated information about messages sent and received, in the case of a timed mix, where all messages are reordered and sent each period of time.
- The method is restricted, at this moment, to threshold mixing with a fixed batch size or, alternatively, to a timed mix, where all messages received in a fixed time period are relayed randomly, reordered with respect to their receivers.
- No restriction is made from before about the number of friends any user has nor about the distribution of messages sent. Both are considered unknown.
- The attacker controls all users in the system. In our real data application, we aim at all email users of a domain sent and received within this domain.

The method introduced in this work allows one to address these general settings in order to derive conclusions about the relationships between users. Contrary to other methods in the literature, there are no restrictions about user relationships (number of friends, distribution of messages), and therefore, it can be used in a wider context. Furthermore, our proposition is new in the methodological sense: this is a novel approach to the problem, by means of contingency table setting and the extraction of solutions by sampling.

In an email context, this attack can be used if the attacker has access, at regular time intervals, to the information represented by the number of messages received and the number of messages sent for each user, in a closed domain or intranet, where all users are controlled. This situation can also be extended to mobile communications or social networks and could be used, for example, in the framework of police communication investigations.

## 5. Marginal Information and Feasible Tables

**Figure 2.**
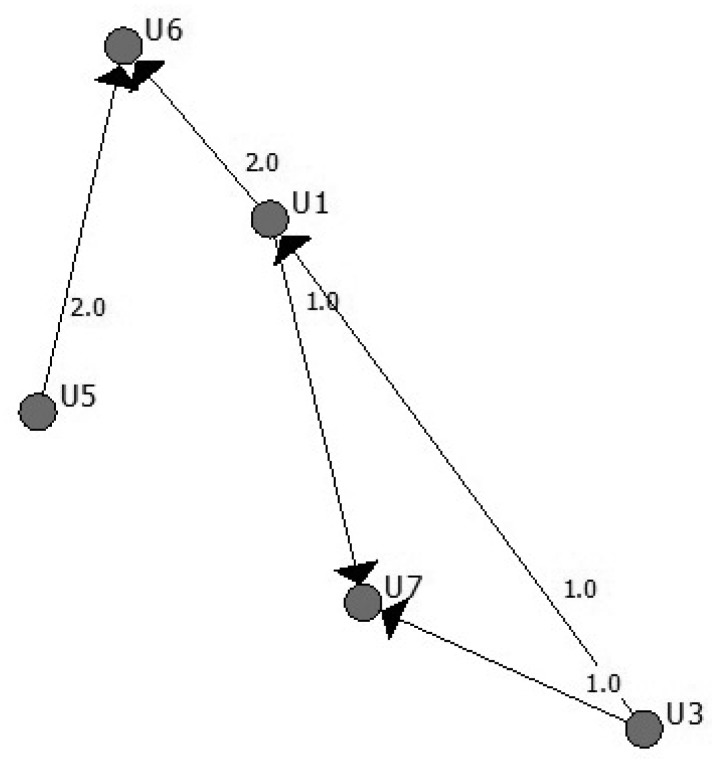
Graphical representation of one round.

The attacker obtains, in each round, information about how many messages each user sends and receives. Usually, the sender and receiver set is not the same, even if some users are senders and also receivers in some rounds. Furthermore, the total number of users of the system N is not present in each round, since only a fraction of them are sending or receiving messages. Figure 2 represents a round with only six users.

The information of this round can be represented in a contingency table (see Table 1), where the element (*i, j*) represents the number of messages sent from user *i* to user *j*:

The attacker only sees the information present in the aggregated marginals, which means, in rows, the number of messages sent by each user, and in columns, the number of messages received by each user. In our example, only the sending pairs of vectors (U1 U3 U5) (3 2 2) and receiver pairs of vectors (U1 U6 U7) (1 4 2) are known.

There are many possible tables that can lead to the table with the given marginals that the attacker is seeing, making it impossible, in most cases, to derive direct conclusions about relationships. The feasible space of the tables' solution of the integer programming problem can be very large. In the example, there are only 16 possible different solutions and only one true solution.

Solutions (feasible tables) can be obtained via algorithms, such as the branch and bound algorithm or other integer programming algorithms. In general they do not guarantee covering evenly all possible tables/solutions, since they are primarily designed to converge to one solution. The simulation framework presented in this article allows us to obtain a large quantity of feasible tables (in the most problematic rounds, it takes approximately three minutes to obtain one million feasible tables). In many of the rounds with a moderate batch size, all feasible tables are obtained.

An algorithm that takes into account the information contained over all of the rounds retrieved is developed in the next section.

## 6. Statistical Disclosure Attack Based on Partial Information

The main objective of the algorithm we propose is to derive relevant information about the relationship (or not) between each pair of users. The information obtained by the attacker is the marginal sums, by rows and columns, of each of the rounds 1, …, *T*, where *T* is the total number of rounds. Note that in each round, the dimension of the table is different, since we do not take into account users that are not senders (row marginal = 0), nor users that are not receivers (column marginal = 0). We say element (*i, j*) is “present” at one round if the *i* and *j* corresponding marginals are not zero. That means that user *i* is present in this round as the sender and user *j* is present as the receiver.

A final aggregated matrix *A* can be built, summing up all of the rounds and obtaining a table with all messages sent and received from each user for the whole time interval considered for the attack. Each element (*i, j*) of this final table would represent the number of messages sent by *i* to *j* in total. Although the information obtained in each round is more precise and relevant (because of the lower dimension and combinatoric possibilities), an accurate estimate of the final table is the principal objective, because a zero in elements (*i, j*) and (*j, i*) would mean no relationship between these users (no messages sent from *i* to *j* nor from *j* to *i*). A positive number in an element of the estimated final table would mean that some message is sent in some round, while a zero would mean no messages are sent in any round, that is, no relationship.

We consider all rounds as independent events. The first step is to obtain the higher number of feasible tables that is possible for each round, taking into account time restrictions. This will be the basis of our attack. In order to obtain feasible tables we use Algorithm 1, based on [22]. It consists of filling the table column by column and computing the new bounds for each element before it is generated.



**Algorithm 1**


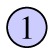
Begin with column one, row one:Generate *n*_11_ from an integer uniform distribution in the bounds according to Equation (1), where *i* = 1*, j* = 1.Let *r* be the number of rows.

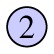
For each row element *n_k_*_1_ in this column, if row elements until *k* — 1 have been obtained, new bounds for *n_k_*_1_ are according to Equation (1):
(1)$\max\left(0,\left(n_{+1}-\sum_{i=1}^{k-1}n_{i1}\right)-\sum_{i=k+1}^{r}n_{i+}\right)\leq n_{k1}\leq \min\left(n_{k+},n_{+1}-\sum_{i=1}^{k-1}n_{i1}\right)$The element *n_k_*_1_ is then generated by an integer uniform in the fixed bounds.

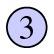
The last row element is automatically filled, since the lower and upper bounds coincide, letting *n*_(*k*+1)+_ = 0 by convenience.

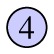
Once this first column is filled, the row margins *n_i_*_+_ and total count *n* are actualized by subtraction of the already fixed elements, and the rest of the table is treated as a new table with one less column.


The algorithm fixes column by column until the whole table is filled.

The time employed depends on the complexity of the problem (number of elements, mean number of messages). In our email data, even for a large number of elements, this has not been a problem. For large table sizes in our applications, it takes approximately 3 min to obtain one million feasible tables in rounds with 100 cells and 10 on a PC with Intel processor 2.3 GHz and 2 GB RAM.

Repeating the algorithm as it is written for each generated table does not lead to uniform solutions, that is some tables are more probable than others due to the order used when filling columns and rows. Since we must consider *a priori* all solutions for a determined round equally possible, two further modifications are made: (i) random reordering of rows and columns before a table is generated; and (ii) once all tables are generated, only distinct tables are kept to make inferences. These two modifications have resulted in an important improvement of the performance of our attack, lowering the mean misclassification rate to about a 20% in our simulation framework.

Deciding the number of tables to be generated poses an interesting problem. Computing the number of distinct feasible tables for a contingency table with fixed marginals is still an open problem that has been addressed via algebraic methods [23] and by asymptotic approximations [24], but in our our case, the margin totals are small and depend on the batch size; therefore, it is not guaranteed that asymptotic approximations hold. The best approximation so far to count the feasible tables is to use the generated tables.

Chen *et al.* [22] show that an estimate of the number of tables can be obtained by averaging over all of the generated tables the value $\frac{1}{q(T)}$ according to the Algorithm 2.


**Algorithm 2**


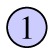
*q*(*T*) is the probability of obtaining the table *T* and is computed iteratively, imitating the simulation process according to Equation (2).

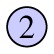
*q*(*t*_1_) is the probability of the actual values obtained for Column 1, obtained by multiplying the uniform probability for each row element in its bounds. *q*(*t*_2_ | *t*_1_) and subsequent terms are obtained in the same way, within the new bounds restricted to the precedent columns fixed values:
(2)$$q(T)=q(t_{1})q(t_{2}|t_{1})q(t_{3}|t_{1},t_{2})\ldots q(t_{c}|t_{1},t_{2},\ldots ,t_{c-1})$$


The number of feasible tables goes from moderate values, such as 100,000, that can be easily addressed, getting all possible tables via simulation, to very high numbers, such as 10^13^. Generating all possible tables for this last example would take, with the computer we are using, a Windows 7 PC with 2.3 GHz and 4 GB RAM, at least 51 days. The quantity of feasible tables is the main reason why it is difficult for any deterministic intersection-type attack to work, even with low or moderate user dimensions. Statistical attacks need to consider the relationships between all users to be efficient, because the space of solutions for any individual user is dependent on all other users' marginals. Exact trivial solutions can be, however, found at some time in the long run, if a large number of rounds are obtained.

In our setting, we try to obtain the largest number of tables that we can, given our time restrictions, obtaining a previous estimate of the number of feasible tables and fixing the highest number of tables that can be obtained for the most problematic rounds. However, an important issue is that once a somewhat large number of tables is obtained, good solutions depend more on the number of rounds treated (time horizon or total number of batches considered) than on generating more tables. In our simulations, there is generally a performance plateau in the curve that represents the misclassification rate *versus* the number of tables generated, since a sufficiently high number of tables is reached. This minimum number of tables to be generated depends on the complexity of the application framework.

The final information obtained consists of a fixed number of generated feasible tables for each round. In order to obtain relevant information about relationships, there is a need to fix the most probable zero elements. For each element, the sample likelihood function at zero *f̂* (*X* | *p_ij_* = 0) is estimated. This is done by computing the percent of tables with that element being zero in each round that the element is present and multiplying the estimated likelihood obtained in all of these rounds (the element will be zero for the final table if it is zero for all rounds).

If we are estimating the likelihood for the element (*i, j*) and are generating *M* tables per round, we use the following expressions:

$n_{t}^{\left(i,j\right)}$ = the number of tables with element (*i, j*) = 0 in round t.
*N_present_* = the number of rounds with element (*i, j*) present.
*X* = the sample data, given by marginal counts for each round.

(3) $$\text{log}\left(\hat{f}\left(X|p_{ij}=0\right)\right)=-N_{\text{present}}\log(M)+\sum_{t=1,(i,j)\text{present}}^{T}\log\left(n_{t}^{\left(i,j\right)}\right)$$

Final table elements are then ordered by the estimated likelihood at zero, with the exception of elements that were already trivial zeros (elements that represent pair of users that have never been present at any round).

Elements with the lowest likelihood are then considered candidates to insert as a “relationship”. The main objective of the method is to detect accurately:

1. cells that are zero with a high likelihood (no relationship *i* → *j*);
2. cells that are positive with high likelihood (relationship *i* → *j*).

In our settings the likelihood values at *p_ij_* = 0 are bounded in the interval [0, 1]. Once these elements are ordered by most likely to be zero to less, a classification method can be derived based on this measure. A theoretical justification of the consistency of the ordering method is given below.

### Proposition 1

Let us consider, a priori, that for any given round k, all feasible tables, given the marginals, are equiprobable.

*Let p_ij_ be the probability of element* (*i, j*) *being zero at the final matrix A, which is the aggregated matrix of sent and received messages over all rounds. Then, the product of the proportion of feasible tables with x_ij_* = 0 *at each round, Q^ij^ leads to an ordering between elements, such that if Q^ij^* > *Q^i′j′^; then, the likelihood of data for p_ij_* = 0 *is bigger than the likelihood of data for p_i′j′_* = 0.

#### Proof

If all feasible tables for round *k* are equiprobable, the probability of any feasible table is $p_{k}=\frac{1}{\#{\left[X\right]}_{k}}$, where # [*X*]*_k_* is the total number of feasible tables in round *k*.

For elements with *p_ij_* = 0, it is necessary that *x_ij_* = 0 for any feasible table. The likelihood for *p_ij_* = 0 is then:

$$P\left({\left[X\right]}_{k}\;|\;p_{ij}=0\right)=\frac{\#{\left[X\;|\;x_{ij}=0\right]}_{k}}{\#{\left[X\right]}_{k}}$$

where # [*X* | *x_ij_* = 0]*_k_* denotes the number of feasible tables with the element *x_ij_* = 0.

Let *k* = 1, …, *t* independent rounds. The likelihood at *p_ij_* = 0, considering all rounds, is:

$$Q^{ij}=\prod_{k=1}^{t}P\left({\left[X\right]}_{k}\;|\;p_{ij}=0\right)=\prod_{k=1}^{t}\frac{\#{\left[X\;|\;x_{ij}=0\right]}_{k}}{\#{\left[X\right]}_{k}}$$

and the log likelihood:

$$\log\left(Q^{ij}\right)=\sum_{k=1}^{t}\log\left(\#{\left[X\;|\;x_{ij}=0\right]}_{k}\right)-\sum_{k=1}^{t}\log\left(\#{\left[X\right]}_{k}\right)$$

Then, the proportion of elements with *x_ij_* = 0 at each round leads to an ordering between elements, such that if *Q^ij^* > *Q^i′j′^*, then the likelihood of data for *p_ij_* = 0 is bigger than the likelihood of data for *p_i′j′_* = 0.

Our method is not based on all of the table solutions, but on a consistent estimator of *Q^ij^*. For simplicity, let us consider a fixed number of *M* sampled tables at every round.

### Proposition 2

*Let* ${\left[X\right]}_{k}^{1},\ldots ,{\left[X\right]}_{k}^{M}$ *be a random sample of size M of the total* #[*X*]*_k_ of feasible tables for round k*. *Let* $w_{k}^{\left(i,j\right)}=\frac{\#{\left[X|x_{ij}=0\right]}_{k}^{M}}{M}$ *be the sample proportion of feasible tables with x_ij_* = 0 *at round k*. *Then, the statistic* $q^{ij}=\overset{t}{\underset{k=1}{\text{∏}}}\frac{\#{\left[X|x_{ij}=0\right]}_{k}^{M}}{M}$ *is such that, for any pair of elements* (*i, j*) *and* (*i′, j′*), *q^ij^* > *q^i′j′^ implies, in convergence, a higher likelihood for p_ij_* = 0 *than for p_i′j′_* = 0.

#### Proof

(1) Let # [*X*]*_k_* be the number of feasible tables at round *k*. Let ${\left[X\right]}_{k}^{1},\ldots ,{\left[X\right]}_{k}^{M}$ be a random sample of size *M* of the total #[*X*]*_k_*. Random reordering of columns and rows in Algorithm 1, together with the elimination of equal tables, assures that it is a random sample. Let $\#{\left[X|x_{ij}=0\right]}_{k}^{M}$ be the number of sample tables with element *x_ij_* = 0. Then, the proportion $w_{k}^{\left(i,j\right)}=\frac{\#{\left[X|x_{ij}=0\right]}_{k}^{M}}{M}$ is a consistent and unbiased estimator of the true proportion $W_{k}^{\left(i,j\right)}=\frac{\#{\left[X|x_{ij}=0\right]}_{k}}{\#{\left[X\right]}_{k}}$. This is a known result from finite population sampling. As $M\to \#{\left[X\right]}_{k},w_{k}^{\left(i,j\right)}\to W_{k}^{\left(i,j\right)}$.
(2) Let *k* = 1,…, *t* independent rounds. Then, given a sample of proportion estimators $w_{1}^{\left(i,j\right)},\ldots ,w_{t}^{\left(i,j\right)}$ of $W_{1}^{\left(i,j\right)},\ldots ,W_{t}^{\left(i,j\right)}$, consider the function
  $$f\left(w_{1}^{\left(i,j\right)},\ldots ,w_{t}^{\left(i,j\right)}\right)=\sum_{k=1}^{t}\log\left(w_{k}^{\left(i,j\right)}\right)\;\text{and}\;f\left(W_{1}^{\left(i,j\right)},\ldots ,W_{t}^{\left(i,j\right)}\right)=\sum_{k=1}^{t}\log\left(W_{k}^{\left(i,j\right)}\right).$$

Given the almost sure convergence of each $w_{k}^{\left(i,j\right)}$ to each $W_{k}^{\left(i,j\right)}$ and the continuity of the logarithm and sum functions, the continuous mapping theorem assures convergence in probability, $f\left(w_{1}^{\left(i,j\right)},\ldots ,w_{t}^{\left(i,j\right)}\right)\underset{\to }{P}f\left(W_{1}^{\left(i,j\right)},\ldots ,W_{t}^{\left(i,j\right)}\right)$. Then, $\text{log}(q^{ij})=f\left(w_{1}^{\left(i,j\right)},\ldots ,w_{t}^{\left(i,j\right)}\right)$ converges to $\text{log}(Q^{ij})=f\left(W_{1}^{\left(i,j\right)},\ldots ,W_{t}^{\left(i,j\right)}\right)$. Since the exponential function is continuous and monotonically increasing, applying the exponential function to both sides leads to the convergence of *q^ij^* to *Q^ij^* , so that *q^ij^* > *q^i′j′^* implies, in convergence, *Q^ij^* > *Q^i′j′^* and, then, higher likelihood for *p_ij_* = 0 than for *p_i′j′_* = 0.

Given all pairs of senders and receivers (*i, j*) ordered by the statistic *q^ij^* , it is necessary to select a cut point in order to complete the classification scheme and to decide whether a pair communicates (*p_ij_* > 0) or not (*p_ij_* = 0). That is, it is needed to establish a value *c*, such that *q^ij^* > *c* implies *p_ij_* = 0 and *q^ij^* ≤ *c* implies *p_ij_* > 0.The defined statistic *q^ij^* is bounded in [0, 1], but this is not strictly a probability, so fixing *a priori* a cut-point, such as 0.5, is not an issue. Instead, there are some approaches that can be used:

1. In some contexts (email, social networks), the proportion of pairs of users that communicate is approximately known . This information can be used to select the cut point from the ordering. That is, if about 20% of pairs of users are known to communicate, the classifier would give a value “0” (no communication) to the upper 80% elements (*i, j*), ordered by the statistic *q^ij^* , and a value “1” (communication) to the lower 20% of elements.
2. If the proportion of zeros is unknown, it can be estimated, using the algorithm for obtaining feasible tables over the known marginals of the matrix A and estimating the proportion of zeros by the mean proportion of zeros over all of the simulated feasible tables.

## 7. Performance of the Attack

In this section, simulations are used to study the performance of the attack.

Each element (*i, j*) of the matrix A can be zero (no communication) or strictly positive. The percentage of zeroes in this matrix is a parameter, set *a priori* to observe its influence. In a closed-center email communications, this number can be between 70% and 99% . However, intervals from 0.1 (high communication density) to 0.9 (low communication density) are used here for different practical situations. Once this percentage is set, a randomly chosen percent of elements are set to zero and then are zero for all of the rounds.

The mean number of messages per round for each positive element (*i, j*) is also set *a priori.* This number is related, in practice, to the batch size that the attacker can obtain. As the batch size (or time length interval of the attack) decreases, the mean number of messages per round decreases, making the attack more efficient.

Once the mean number of messages per round is determined for each positive element (*λ_ij_*), a Poisson distribution with mean *λ_ij_, P*(*λ_ij_*), is used to generate the number of messages for each element, for each of the rounds.

External factors, given by the context (email, social networks, *etc.*) that have an effect on the performance of the method are monitored to observe their influence:

1. The number of users: In a network communication context with *N* users, there exist *N* potential senders and *N* receivers in total, so that the maximum dimension of the aggregated matrix A is *N*^2^. As the number of users increases, the complexity of round tables and the number of feasible tables increases, so that it could negatively affect the performance of the attack.
2. The percent of zero elements in the matrix A: These zero elements represent no communication between users. As will be seen, this influences the performance of the method.
3. The mean frequency of messages per round for positive elements: This is directly related to the batch size, and when it increases, the performance is supposed to be affected negatively.
4. The number of rounds: As the number of rounds increases, this is supposed to improve the performance of the attack, since more information is available. One factor related to the settings of the attack method is also studied.
5. The number of feasible tables generated by round: This affects computing time, and it is necessary to study to what extent it is useful to obtain too many tables. This number can be variable, depending on the estimated number of feasible tables for each round .

The algorithm results in a binary classification, where zero in an element (*i, j*) means no relationship of sender-receiver from *i* to *j* and one means a positive relationship of sender-receiver.

Characteristic measures for binary classification tests include the sensitivity, specificity, positive predictive value and negative predictive value. Letting TP be true positives, FP false positives, TN true negatives and FN false negatives:

Sensitivity= $\frac{TN}{TN+FP}$ measures the capacity of the test to recognize true negatives.
Specificity= $\frac{TP}{TP+FN}$ measures the capacity of the test to recognize true positives.
Positive predictive value = $\frac{TP}{TP+FP}$ measures the precision of the test to predict positive values.
Negative predictive value = $\frac{TN}{TN+FN}$ measures the precision of the test to predict positive values.
Classification rate = $\frac{TN+TP}{TN+TP+FN+FP}$ measures the percent of elements well classified.

**Figure 3.**
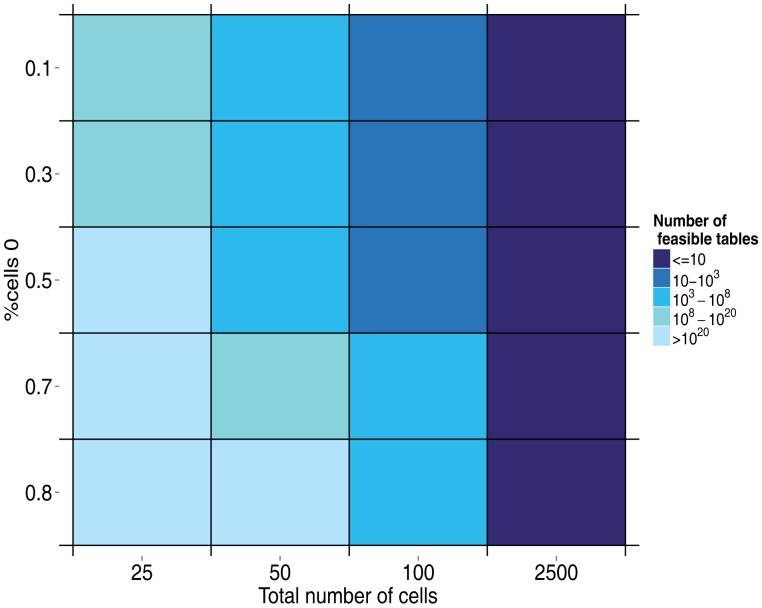
Number of feasible tables per round, depending on % of cells of zero and the total number of cells.

**Figure 4.**
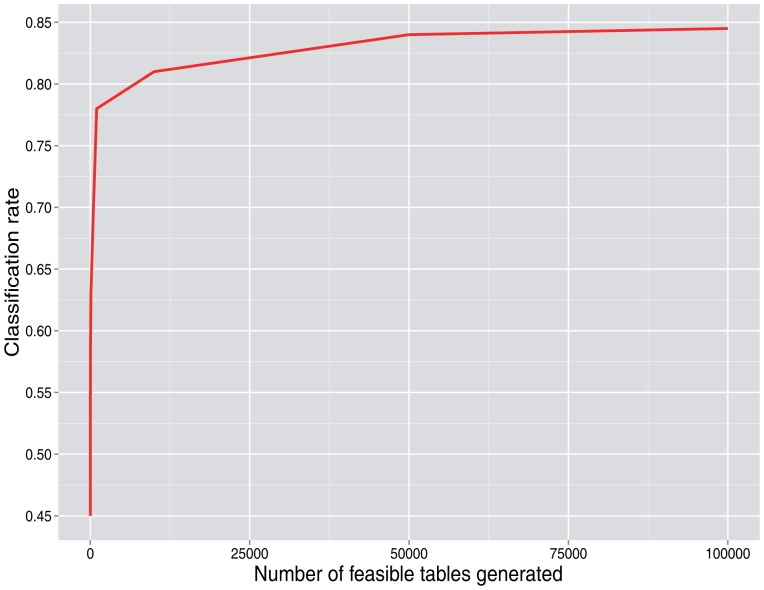
Classification rate as function of the number of feasible tables generated per round.

Figures 3 and 4 show the simulation results. When it is not declared, values of *p*_0_ = 0.7, *λ* = 2, *N* = 50 users and the number of rounds = 100 are used as base values.

Figure 3 shows that as the number of cells (*N*^2^, where *N* is the number of users) increases and the percent of cells that are zero decreases, the number of feasible tables per round increases. For a moderate number of users, such as 50, the number of feasible tables is already very high, greater than 10^20^. This does not have a strong effect on the main results, except for lower values. As can be seen in Figure 4, once a sufficiently high number of tables per round is generated, increasing this number does not lead to significant improvement of the correct classification rate.

**Figure 5.**
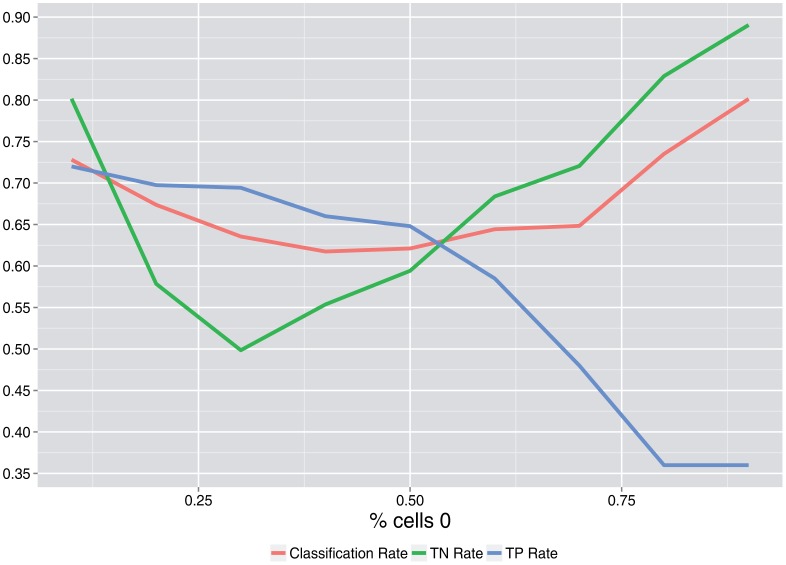
Classification rate, true positive rate and true negative rate *vs*. the percent of cells of zero.

Figure 5 shows that the minimum classification rate is attained at a percent of cells of zero (users that do not communicate) near 0.5. As this percent increases, the true positive rate decreases, and the true negative rate increases.

**Figure 6.**
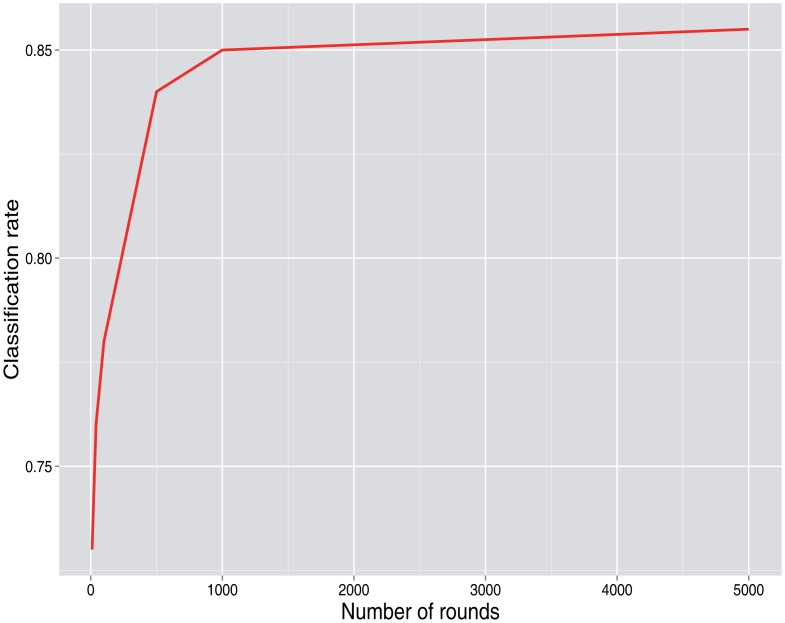
Classification rate *vs*. the number of rounds obtained.

As the attacker gets more information, that is more rounds are retrieved, the classification rate gets better. Once a high number of rounds is obtained, there is no further significant improvement, as is shown in Figure 6.

**Figure 7.**
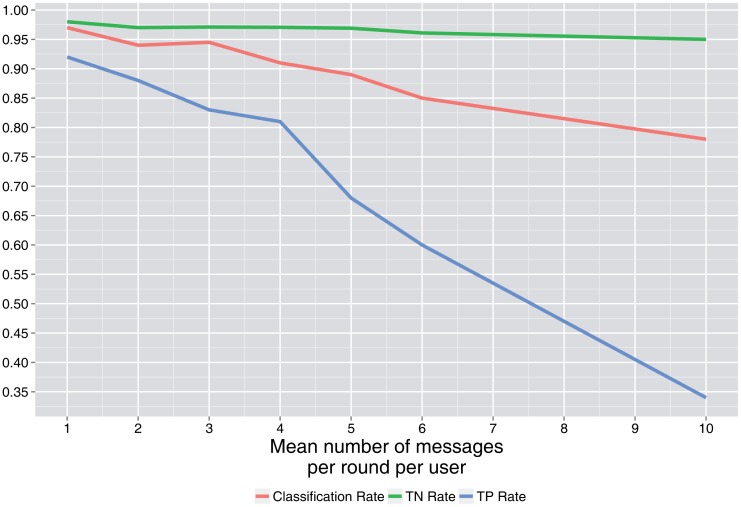
Classification rates *vs*. the mean number of messages per round.

In Figure 7, it is shown that as the number of messages per round (*λ*) for users that communicate increases, the classification rates decrease. This is a consequence of the complexity of the tables involved (more feasible tables). This number is directly related to the batch size, so it is convenient for the attacker to obtain data in small batch sizes and for the defender to group data in large batch sizes, leading to lower latency.

**Figure 8.**
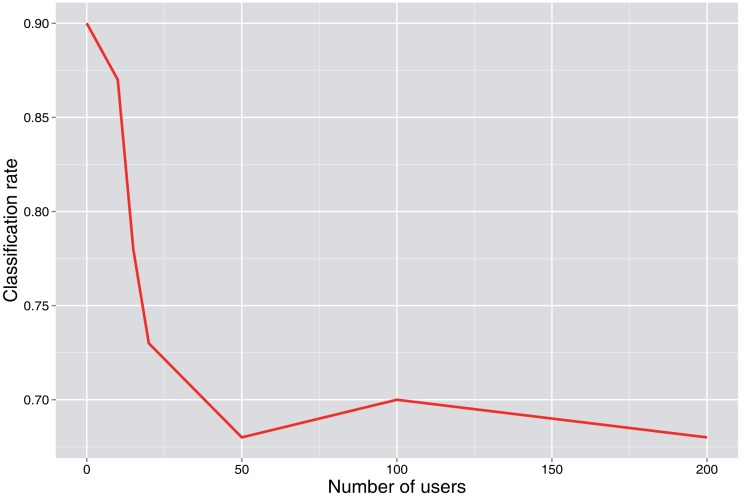
Classification rate *vs*. the number of users.

The complexity of the problem is also related to the number of users, as can be seen in Figure 8, where the classification rate decreases as the number of users increases.

## 8. Conclusions

This work presents a method to detect relationships (or non-existent relationships) between users in a communication framework, when the retrieved information is incomplete. The method can be extended to other settings, such as pool mixes, or situations where additional information can be used. Parallel computing has also been successfully used in order to obtain faster results. The method can also be used for other communication frameworks, such as social networks or peer-to-peer protocols, and for real de-anonymization problems not belonging to the communications domain, such as disclosing public statistical tables or forensic research. More research has to be done involving the selection of optimal cut points, the optimal number of generated tables or further refinements of the final solution, which may be through the iterative filling of cells and cycling the algorithm.

## Figures and Tables

**Table 1.** Example of a contingency table.

**Senders\Receivers**	**U1**	**U6**	**U7**	**Total Sent**
U1	0	2	1	3
U3	1	0	1	2
U5	0	2	0	2
Total received	1	4	2	7
